# Particleboard Production from *Paulownia tomentosa* (Thunb.) Steud. Grown in Portugal

**DOI:** 10.3390/polym15051158

**Published:** 2023-02-24

**Authors:** Bruno Esteves, Pedro Aires, Umut Sen, Maria da Glória Gomes, Raquel P. F. Guiné, Idalina Domingos, José Ferreira, Hélder Viana, Luísa P. Cruz-Lopes

**Affiliations:** 1 Department of Wood Engineering, Polytechnic Institute of Viseu, Av. Cor. José Maria Vale de Andrade, 3504-510 Viseu, Portugal; 2 Centre for Natural Resources, Environment and Society-CERNAS-IPV, Av. Cor. José Maria Vale de Andrade, 3504-510 Viseu, Portugal; 3 Forest Research Centre (CEF), The School of Agriculture, Tapada da Ajuda, 1349-017 Lisbon, Portugal; 4 CERIS, Department of Civil Engineering, Architecture and Georesources, Instituto Superior Técnico, Universidade de Lisboa, Av. Rovisco Pais, 1049-001 Lisbon, Portugal; 5 Department of Food Engineering, Agrarian School of Viseu, Quinta da Alagoa, Estrada de Nelas, Ranhados, 3500-606 Viseu, Portugal; 6 Department of Ecology and Sustainable Agriculture, Polytechnic Institute of Viseu, Av. Cor. José Maria Vale de Andrade, 3504-510 Viseu, Portugal; 7 Centre for the Research and Technology of Agro-Environmental and Biological Sciences (CITAB), University of Trás-os-Montes and Alto Douro (UTAD), Quinta de Prados, 5000-801 Vila Real, Portugal; 8 Department of Environmental Engineering, Polytechnic Institute of Viseu, Av. Cor. José Maria Vale de Andrade, 3504-510 Viseu, Portugal

**Keywords:** young Paulownia, particleboard, density, thermal conductivity, mechanical properties, physical properties, water absorption

## Abstract

Paulownia wood has raised high attention due to its rapid growth and fire resistance. The number of plantations in Portugal has been growing, and new exploitation methods are needed. This study intends to determine the properties of particleboards made with very young Paulownia trees from Portuguese plantations. Single layer particleboards were produced with 3-year-old Paulownia trees using different processing parameters and different board composition in order to determine the best properties for use in dry environments. The standard particleboard was produced at 180 °C and a 36.3 kg/cm^2^ pressure for 6 min using 40 g of raw material with 10% urea-formaldehyde resin. Higher particle size lead to lower-density particleboards, while higher resin contents lead to higher density of the boards. Density has a major effect on board properties with higher densities improving mechanical properties such as bending strength, modulus of elasticity (MOE) and internal bond, lower water absorption but higher thickness swelling and thermal conductivity. Particleboards meeting the requirements for dry environment according to NP EN 312 standard, could be produced with young Paulownia wood with acceptable mechanical and thermal conductivity properties with density around 0.65 g/cm^3^ and a thermal conductivity of 0.115 W/mK.

## 1. Introduction

Wood is a scarce commodity, and neither natural or coppice forests are unlimited reserves, so technological and industrial centers have begun the development of alternative products capable of responding to the growing demand for wood and wood-based panels [1,2,3]. In Portugal, the particleboard industry works almost exclusively with pine wood (*Pinus pinaster*), which is one of the most abundant resources in the Portuguese forest. In recent years, there have been some studies on new species that may have a higher production yield in the short term, as is the case of the Paulownia species [2,4,5,6]. Also several forest and agricultural wastes have been tested for particleboard production; nevertheless, most of these materials have high heterogeneity and seasonal availability [1,4].

The genus Paulownia, belonging to the Family Paulowniaceae, consists of nine species: *P. albiphloea*, *P. australis*, *P. catalpifolia*, *P. elongata*, *P. fargesii*, *P. fortunei*, *P. kawakamii*, *P. taiwaniana*, and *P. tomentosa* and several hybrids such as cotevisa 2, produced in Spain, which is the result of the crossing *Paulownia elongata* × *Paulownia fortunei* [7,8].

Paulownia wood is easily air-dried without severe drying defects. It is more resistant to fires than other fast-growing species due to its high ignition temperature, high water content in the fire season and large leaves. The vessel structure of Paulownia wood is very large and independent, making it difficult for oxygen to be adequately supplied and making this wood difficult to ignite [9]. It has a high resistance-to-weight ratio, a low shrinkage coefficient and does not deform or crack easily [8,10]. The workability and finishing properties of the wood are excellent, but it is considered an underused species [11]. Nevertheless, Paulownia wood has been used for plywood, paper production, veneers, and some objects such as, for example, rice pots, bowls or spoons [12,13]. *Paulownia tomentosa* has shown a very fast growing rate, which permits its utilization in short rotation periods. Fast growing has the downsides of producing low-density wood with poor mechanical properties and, therefore, not being suitable for structural applications. On the other hand, low density also leads to low thermal conductivity, which might be important for thermal insulation boards. This is a great advantage in relation to pine wood since it can allow the elaboration of particleboards with lower density and thermal conductivity. According to Akyildiz and Kol [10], this species has a specific mass of approximately 35 g/cm^3^, but the density can vary according to the age of the tree and plantation location. According to Kim et al. [14], wood grown in Korea has a lower density, around 0.27 g/cm^3^, while wood growth in Portugal has a higher density, around 0.44 g/cm^3,^ possibly due to the slower growth [15]. Even in the same plantation, there are significant differences seen in Paulownia trees planted in Hungary that had a density of 0.28 g/cm^3^ on average but ranged from 0.24 g/cm^3^ to 0.33 g/cm^3^ [16]. On the other hand, *Paulownia tomentosa* × *elongata* planted in Spain, Bulgaria and Serbia presented a similar density of around 0.26 g/cm^3^ [17]. The thermal conductivity of *Paulownia tomentosa* wood has been reported to be between 0.073–0.100 W/mK [18].

Several parameters affect the final properties of particleboards, such as properties of the raw material, like for instance, species, density or particle size and shape; the properties of the resin, such as kind, quality and quantity; and those of the pressing system like pressure, temperature, press-closing time and pressing time [19,20,21,22,23]. The density of the raw material influences the density of the panel and, consequently, its mechanical and physical properties. This relationship is called compaction ratio. Using raw materials with low density is better for the production of medium density panels since they have an adequate compaction ratio (1.3) which allows good contact area between particles during pressing, leading to a good bonding [24,25]. Generally, higher particle sizes have been reported to lead to lower density due to the lower compaction ratio of coarse particles in relation to finer particles, as mentioned by Cosereanu et al. [20] for particleboard made with sunflower seed husk. Ferrandez-Garcia [26], with boards made from Washingtonia Palm rachis with citric acid, Hegazy and Ahmed [27], with particleboards manufactured from Date Palm fronds or Osarenmwinda, and Nwachukwu [28], with rice husk particleboard, all obtained a similar decrease for bigger particles, while Farrokh Payam et al. [29] obtained an increase followed by a decrease. However, in accordance with Bazetto et al. [24], who tested the particle size effect on bamboo particleboard properties, no significant influence was found on board density, but bending strength and modulus of elasticity (MOE) decreased. These results are possibly because of the narrow range of particle size used (between 0.210 and 0.420 mm) in the study. The shape of the particles also influences the particleboard properties. For instance, the concave particles of sunflower seed husk decrease the compaction of the particleboard, negatively affecting the board properties [20]. The spreading or mat density is related to particle dimensions. As the particle thickness and width increase, the mat density also increases while the particle length has the opposite effect [21]. Another study showed that the longer the particles, the higher the mechanical properties (IB, and MOR, respectively) and the lower the water adsorption and thickness swelling [30]. The type of resin has an obvious influence on board properties. For example, for particleboards made with Asian bamboo and three different resins, melamine formaldehyde (MF), melamine urea phenol formaldehyde (MUPF) and phenol formaldehyde (PF), Malanit et al. [31] reported that melamine formaldehyde obtained the best results. Also, particleboards produced using isocyanate resins result in better mechanical properties and dimensional stability than those produced using urea formaldehyde resins [32]. Higher amounts of resin generally improve the board properties, mainly thickness swelling and mechanical properties as stated before by Rathke et al. [33] in a study of particleboards made from willow, poplar, and locust, or by Nemli et al. [23] in a study of particleboard panels consisting of 45% beech (*Fagus orientalis*), 35% pine (*Pinus nigra*) and 20% poplar (*Populus nigra*), or even by Arabi et al. [34] who reported that higher resin content lead to increased mechanical properties for single-layer particleboards made from poplar wood. Increased hot pressing time and temperature has often been mentioned to improve the mechanical properties of the particleboards, like, for instance, for Asian bamboo [31] or Jatropha Fruit Hulls treated in acidic conditions, particleboard made from sorghum bagasse as reported by Iswanto et al. [35,36] or particleboard made from recycled particles bonded with a new natural adhesive composed of tannin and sucrose [37].

The density of the panel is probably the most important factor since it considerably affects both physical and mechanical properties of the panels [5,27,34,38]. Several studies have shown that boards with higher density have higher thickness swelling but lower water absorption and better mechanical properties such as MOR, MOE and Internal bond strength [11,19,38,39,40].

This work intended to determine for the first time, the feasibility of using very young trees from *Paulownia tomentosa* wood with only 3 years of growth, grown in Portugal, for single-layer particleboard production. Its fast-growing rate would allow a sustainable forest management since the wood can be harvested sooner than traditional wood species.

## 2. Materials and Methods

### 2.1. Sampling and Material Preparation

Young age Paulownia wood (*Paulownia tomentosa* (Thunb.) Steud.) used in the work was harvested in an experimental plantation field at the Agriculture High School of Polytechnic Institute of Viseu, cultivated under the Carbo Energy and Biomass coppice Project (PROJ/CI&DETS/CGD/0008).

**Figure 1:**
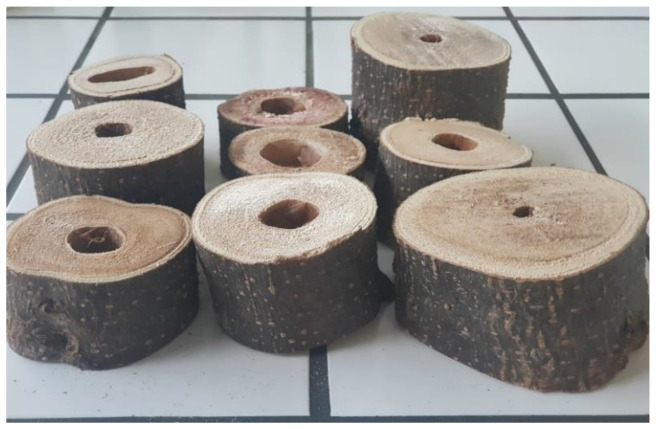
*Paulownia tomentosa* samples after being cut.

In this study, 3-year-old Paulownia wood was cut into small logs (Figure 1) and air dried until it reached a moisture content of around 12%. Afterwards, the logs were debarked, turned into chips with a chisel and milled into particles in a knife Retsch SMI mill (Haan, Germany), followed by sifting in a Retsch AS200 (Haan, Germany) sifter for 20 min at 50 rpm. Four distinct fractions (<0.25 mm; 0.25–0.4 mm; 0.4–1.18 mm and >1.7 mm) were obtained. After this separation, the particles were dried until they reached a final moisture content of between 3 and 4%.

### 2.2. Preparation of Particleboard

**Figure 2:**
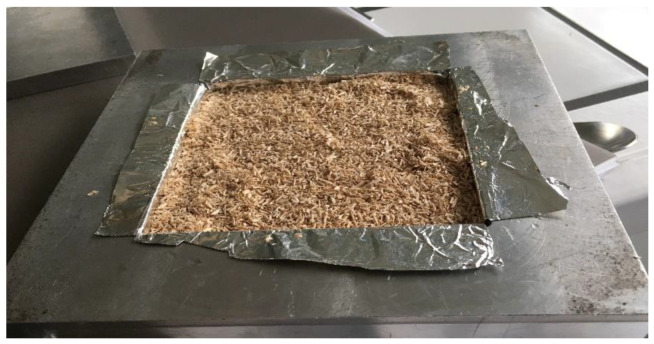
Stainless steel mold with ready-to-press mixture.

For the formation of single-layer particleboards, the samples were mixed with a UF resin with 64% resin solids content (EuroResinas—Indústrias Químicas S. A., Sines, Portugal) in an Ika Ost Basic mixer at 750 rpm. In the mixing process, the resin was slowly added so that it was evenly distributed. The mattress was formed in a square stainless-steel mold of 100 mm × 100 mm (Figure 2) lined with aluminum foil to ensure that the mixture did not adhere to the mold during pressing.

The samples were pressed in a Carver press 3889CE (Wabash, IN, USA) with a standard temperature of 180 °C, 10% resin content and a 36.3 kg/cm^2^ pressure for 6 min. After this step, the mold was removed from the press, and the board cooled until it was possible to remove the foil. The final weight and dimensions of the board were determined in an analytical scale and with the aid of a caliper. In each board, an average of 40 g of raw material was used with 10% urea-formaldehyde resin.

**Figure 3:**
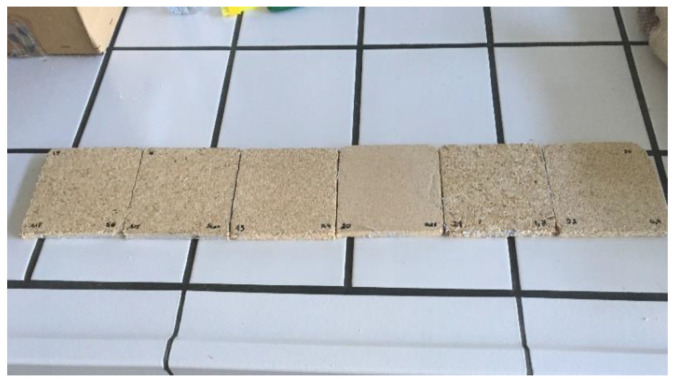
*Paulownia tomentosa* particleboard boards.

In order to test the influence on particleboard properties, the percentage of resin used varied between 8–12% while particle size varied between 0.25–0.4 mm; 0.4–1.18 mm and >1.7 mm. These variations allowed us to more accurately evaluate their influence on the quality of particleboard boards, which are visible in Figure 3.

### 2.3. Particleboard Testing

Density was determined for particleboard conditioned at 20 °C and 65% relative humidity by weighing and measuring the board dimensions in the three directions (approximately 100 mm × 100 mm × 8 mm).

Before all the tests, particleboard was conditioned at 20 °C and 65% humidity.

Bending strength and modulus of elasticity (MOE) were determined in a universal test machine Servosis I—405/5 using a 3-point bending test according to NP EN 310 standard [41] with some modifications. Each board was cut into 25 mm × 100 mm long samples. The samples were placed on the machine with an 80 mm span and subjected to growing tension with an applied load at a constant rate of cross-head movement so that the maximum load was reached within 60 ± 30 s. Tests were made in triplicate. Bending strength (BS) and modulus of elasticity (MOE) were determined in accordance to:

(1) $$\mathrm{BS}\;(\mathrm{MPa})=\frac{3*F_{max}*l_{1}}{2*b*t^{2}}$$

*l*_1_ is the span in millimeters;

*b* is the width of the test piece, in millimeters;

*t* is the thickness of the test piece, in millimeters;

*F_max_* is the maximum load, in Newtons.

(2) $$\mathrm{MOE}\left(\mathrm{MPa}\right)=\frac{\left(F_{2}-F_{1}\right)\times l_{1}}{\left(\alpha_{2}-\alpha_{1}\right)\times 4\times b\times t^{3}}$$

*l*_1_ is the span in millimeters;

*b* is the width of the test piece, in millimeters;

*t* is the thickness of the test piece, in millimeters;

*F*_2_ − *F*_1_ is the increment of load on the straight-line portion of the load-deflection curve, in N;

*a*_2_ − *a*_1_ is the increment of deflection at the mid-length of the test piece (corresponding to *F*_2_ − *F*_1_) in millimeters.

Internal bond Measurement was done in accordance with NP EN 319 [42]. Samples with 50 × 50 mm were glued in aluminum blocks with the same dimensions. A tension load with a loading speed of approximately 2 mm = min, adjusted so that the maximum load was reached within 60 ± 30 s, was applied vertically to the board face. The maximum load (*P*) supported by the sample with dimensions (*b* × *L*) was recorded, and internal bond was calculated according to the following equation:

(3) $$\mathrm{Internal}\;\mathrm{bond}\;(N/{\mathrm{mm}}^{2})=\frac{P}{b\times L}$$

Water absorption (WA) and thickness swelling (TS) were determined in accordance to NP EN 317 [42] by conditioning the samples at 65% relative humidity and 20 °C prior to water immersion. The samples were kept vertically in water at 20 °C and pH 7. After 24 h, the samples were removed from the water bath, wiped with an absorbing paper, weighed and measured with a digital caliper on the middle of the sample. Water absorption and thickness swelling were determined in accordance with Equations (3) and (4):

(4) $$\mathrm{Water}\;\mathrm{absorption}=\frac{m_{f}-m_{i}}{mi}\times 100$$

(5) $$\mathrm{Thickness}\;\mathrm{swelling}=\frac{t_{f}-t_{i}}{t_{i}}$$

where *f* means final after the soaking period, and *i* means initial conditions (65% RH and 20 °C).

The thermal conductivity (λ, W/mK) was determined by means of a modified transient pulse method (MTPS), following the ASTM-D-5334 [43], ASTM D 5930 [44] and EN 22007-2 [45] testing procedures, using an ISOMET 2114 portable hand-held heat transfer analyzer, from Applied Precision Enterprise. Each measurement took approximately 25 min and was performed with a surface measurement probe placed on top of each sample surface at three different points. The surface probe has a constantly powered resistor heater that imposes a heat impulse on the sample in thermal equilibrium with the surrounding environment [43,44].

### 2.4. Statistical Analysis

All the tests with the particle size, resin content and pressing temperature were made in triplicate. Error bars represent ±σ (standard deviation). A linear regression model was used to test the relation between the independent variables. The coefficient of variation was also determined to test the dispersion of data points around the mean.

## 3. Results and Discussion

There are many factors influencing the board properties, such as board constitution (material, size and shape of particles, resin kind and content) or board production parameters (temperature, pressure or pressing time) [21,22,23,34,36]. All these parameters influence the board density, which in turn influences mechanical properties like bending strength and modulus of elasticity, internal bond, and water absorption and thickness swelling. Density has also proven to be the most important property in relation to the thermal conductivity of particleboards. Therefore, several parameters were studied in order to obtain the lowest conductivity possible while fulfilling the mechanical and physical properties requirements of NP EN 312 [46].

### 3.1. Influence of Constitution on Board Properties

The results showed that the initial wood density was around 0.42 g/cm^3^ and that the final densities of the boards varied between 0.39 g/cm^3^ and 0.92 g/cm^3^, depending on pressing parameters or board factors such as particle size or percentage of resin used. These variations will be discussed in detail in this section. Earlier studies suggested that a board density lower than the wood density is not advisable; therefore, the boards should have the lowest density compatible with the minimum requirements for particleboard [40], and the board density has to be higher than the wood species density so that there is a good inter-particle contact or else the resin would polymerize in the void spaces, leading to poor bonding [40].

**Figure 4:**
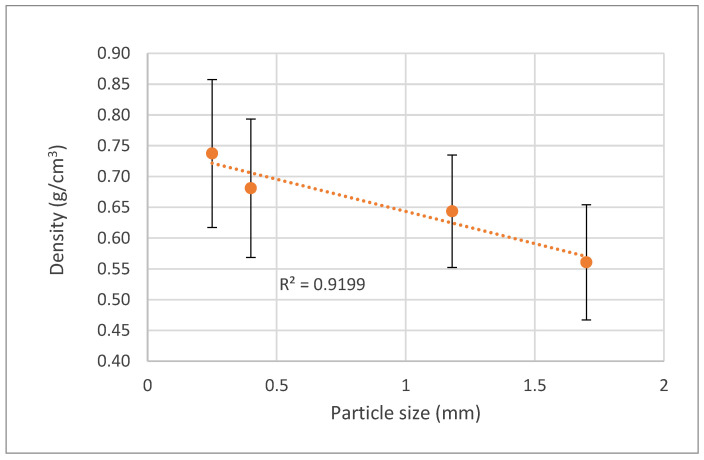
Correlation between board density variation and raw wood particle size.

The size of the wood fractions has proven to be one of the most important parameters in the final density of the particleboards. According to Figure 4, it is possible to verify that, at similar conditions, when larger particles are used, lower-density boards are produced. The smaller fraction (0.25–0.4 mm) has an average density of 0.75 g/cm^3^, while in the largest fraction (1.7–2 mm), it is around 0.55 g/cm^3^. The coefficient of determination (R^2^ = 0.92) is considered good, considering that the linear regression model explains 92% of the variance obtained. The standard deviation between samples is not very small, but the coefficient of variation ranges from 13% to 18%, which means that there is a low-to-medium dispersion of the results. This shows that, since the final objective is the production of lower-density boards to obtain lower thermal conductivity, particles of larger dimensions should be used if they do not significantly impair the panel properties.

The increase in board density with decreasing particle size is probably due to higher compaction when using smaller particles. Similar results have been reported before for single-layer particleboard made from sunflower seed husks [20]. Particle size has been mentioned before as one of the most important factors that can be used to influence the physical and mechanical properties of particleboards [47]. These authors stated that, by increasing the length of the particles, the bending strength and MOE increases, but, on the other hand, internal bond decreases [34,48,49].

**Figure 5:**
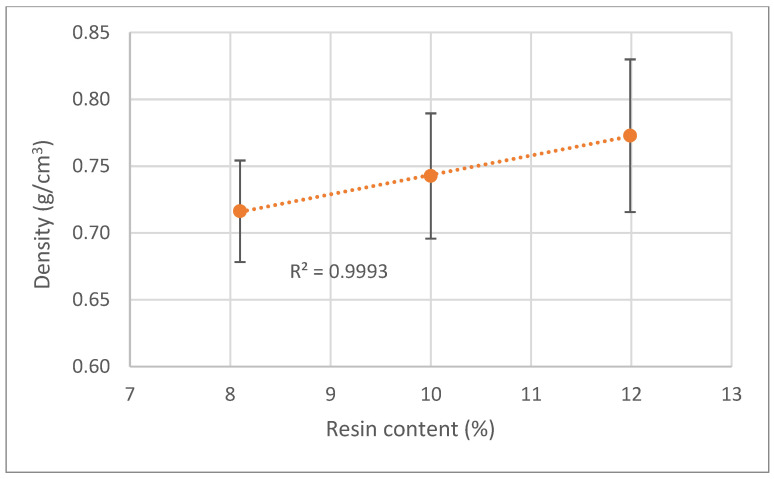
Correlation between board density variation and resin content.

It is not only the particle size that can affect the board density. Figure 5 presents the effect of resin content on board density. Results show that a higher percentage of resin leads to a higher density of the board, which was expected since wood density was around 0.42 g/cm^3,^ and the UF resin density is much higher 1.2–1.3 g/cm^3^. The relationship between resin content and density appears to be linear, at least between 8–12% resin content. Similar results were presented before by Rathke et al. [33] that tested the effects of alternative raw materials and varying resin content on the mechanical properties of particleboards. Nevertheless, higher resin contents increase the price of the boards and the formaldehyde release during use. Therefore, a high resin content is to be avoided. Once again, the determination coefficient is very high, R^2^ = 0.999, and the dispersion of the results is low with a maximum coefficient of variation of 7%.

**Figure 6:**
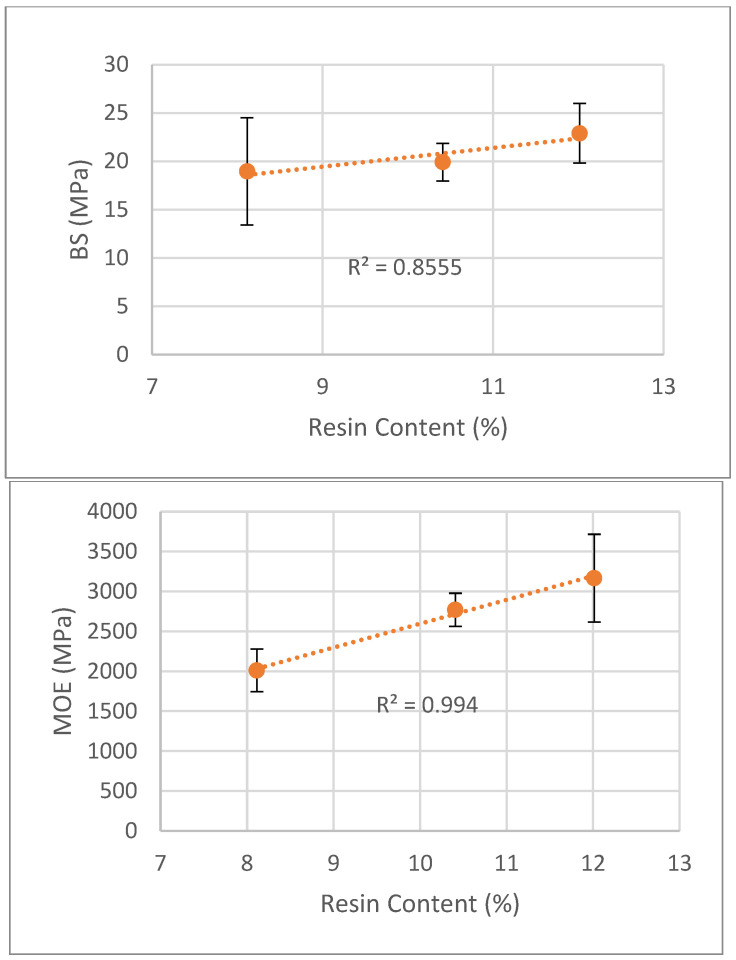
Correlation between bending strength and MOE variation and resin content.

In order to study the resin content effect on board properties with the same density, a target density of 0.50 g/cm^3^ was used. At similar density, a higher resin content leads to better mechanical properties, with a higher bending strength and modulus of elasticity, as can be seen in Figure 6. Bending strength increased from around 19 MPa to 23 MPa for 8–12% resin content which represents a 21% increase, while MOE changed from 2000 MPa to 3170 MPa (58% increase).

Similar results were presented before by, for instance, Lehmann [50] with 2, 4 and 8 percent resin solids of urea-formaldehyde resin that obtained increases in both bending strength and modulus of elasticity, by Nemli et al. [51] with kiwi (*Actinidia sinensis* Planch.) pruning particleboard or by Rathke et al. [33] with different wood species. Arabi et al. [34] stated that MOR and MOE increased with resin content, and that an exponential function can better describe the simultaneous effect of slenderness and resin content than the linear equation. On the other hand, Kimoto et al. [52] tested resin contents from 8–15% and stated that only minor improvements were found in strength with 15% resin content compared to 10%. In relation to the determination coefficient for the relation between both bending strength and modulus of elasticity with resin content, the first was only 0.856 and the second was 0.994. On the other hand, the standard deviations that seems very small due to the scale, range from 5% to 25% which is in the limit of medium dispersion.

**Figure 7:**
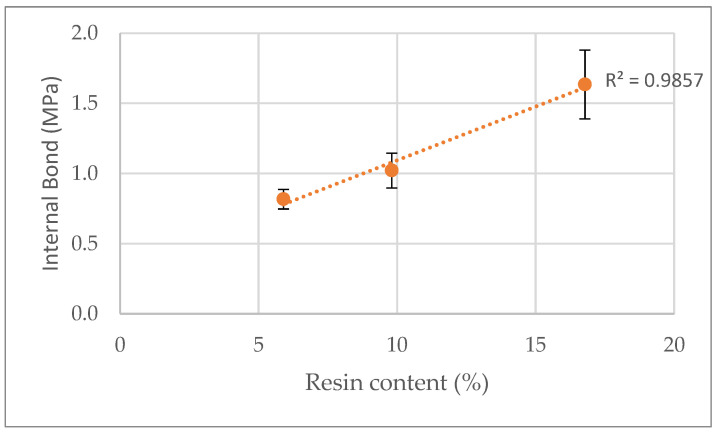
Correlation between internal bond variation and resin content.

Internal bond (Figure 7) increases with the increase in resin content from around 0.7 MPa for 6% resin content to more than 1.5 MPa for 17% resin content. Similarly, Nemli et al. [23] reported that internal bond increased from 0.483 to 0.648 MPa for commercially produced particleboard panels with 45% beech (*Fagus orientalis*), 35% pine (*Pinus nigra*) and 20% poplar (*Populus nigra*) with 8–10% and 10–12% resin content. The same was reported by Dai et al. [53] for strand boards. In relation to internal bond, the coefficient of determination is 0.986, and the coefficient of variation reaches a maximum of 20% for the highest resin content; therefore, the dispersion of the results can be considered between small to medium.

**Figure 8:**
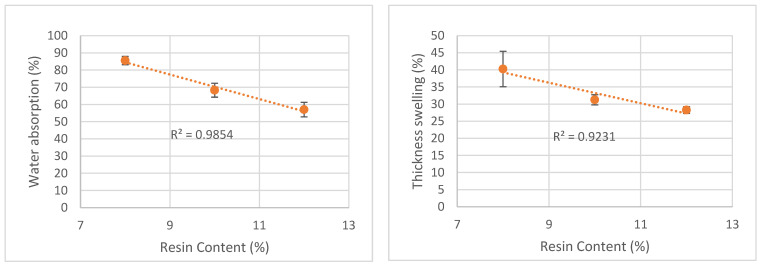
Correlation between water absorption and thickness swelling variation and resin content.

Water absorption variation and thickness swelling with resin content are presented in Figure 8. Results show that higher resin content leads to lower water absorption and lower thickness swelling. Similar results were reported before by Sekaluvu et al. [54] with particleboards made with Maize Cob. These authors observed an increase in MOE and MOR and a decrease in water absorption and thickness swelling for higher resin contents. Similarly, Ashori and Nourbakhshb [55], who studied the effect of press cycle time and resin content on the physical and mechanical properties of particleboard panels made from Date palm, Eucalyptus, Mesquite and Saltcedar, concluded that higher resin content decreased the thickness swelling for all the studied materials. Nemli et al. [23] also reported a lower thickness swelling for higher resin content.

**Figure 9:**
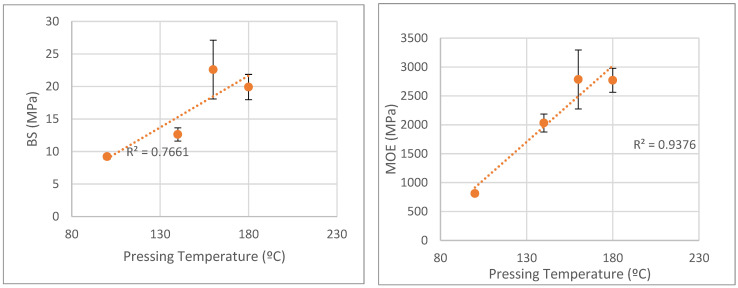
Correlation between bending strength and MOE variation with pressing temperature.

The pressing temperature presented in Figure 9 was tested for a target density of 0.50 g/cm^3^ with 10% UF resin content. The pressing time used was 6 min at 36.3 kg/cm^2^ pressure. Generally, mechanical properties increased with the pressing temperature. Results showed that a temperature of at least 160 °C was needed in order to obtain good mechanical properties, which could be due to the low pressing time. A higher temperature is needed for the core temperature to reach the degree required to cure the resin. Bending strength presented higher values for 160 °C than for 180 °C, although values presented high standard deviations, as seen in Figure 6. Similar results were presented before for particleboard made from sorghum bagasse [36], where mechanical properties increased for higher pressing temperature, with the exception of 180 °C in relation to 170 °C. This could be due to some thermal degradation at higher temperatures. Similarly, Iswanto et al. [35,36] reported increased mechanical properties with a higher pressing temperature of particleboard made from Jatropha Fruit Hulls treated in acidic conditions and particleboard made from sorghum bagasse. However, the temperatures tested were lower, ranging from 110 °C to 130 °C. Malanit et al. [31] reported that higher pressing temperature resulted in higher mechanical strength of UF bonded Asian Bamboo particleboards, for pressing temperatures ranging from 150 °C to 210 °C but using three different resins (melamine formaldehyde, melamine urea-formaldehyde and phenol formaldehyde). The variation between pressing temperature and BS and MOE does not seem to be linear with determination coefficients of 0.766 and 0.938. The dispersion of the results goes from very small for 100 °C to medium for 160 °C.

### 3.2. Influence of Density on Board Properties

**Figure 10:**
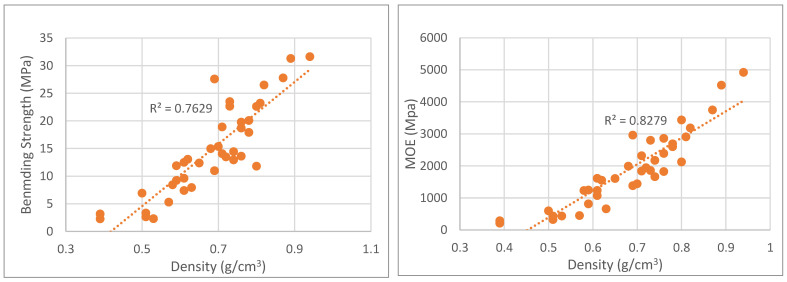
Correlation between bending strength and MOE variation with particleboard density.

**Figure 11:**
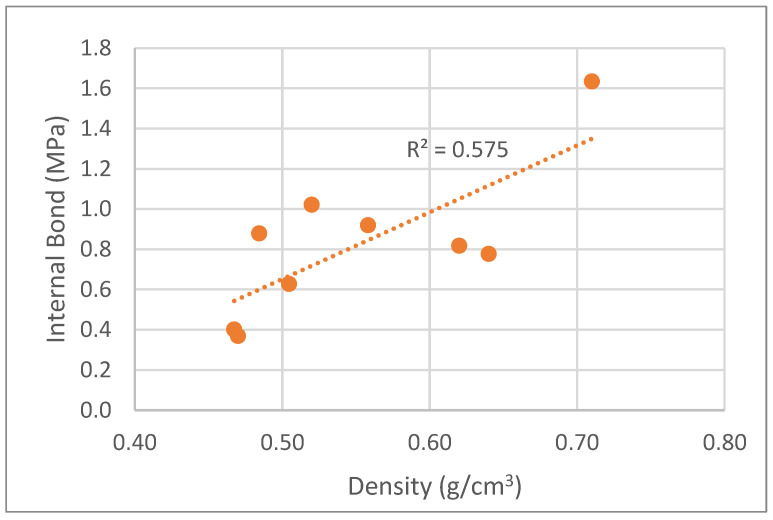
Correlation between internal bond variation with particleboard density.

**Figure 12:**
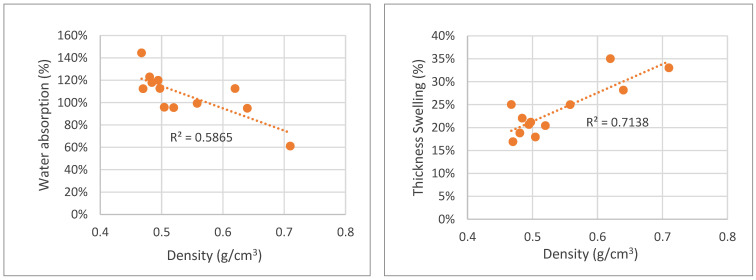
Correlation between water absorption and thickness swelling variation with particleboard density.

**Figure 13:**
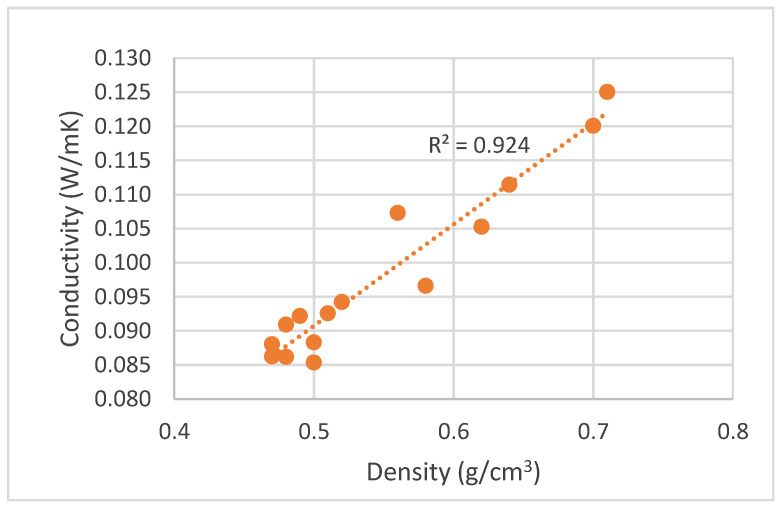
Correlation between conductivity variation and particleboard density.

Density is most likely the major factor when addressing the board properties. Figure 10, Figure 11, Figure 12 and Figure 13 present the variation of mechanical properties (bending strength, MOE and internal bond), water absorption, TS and thermal conductivity with density. The mechanical properties of particleboards increased with the board density. Even with several different parameters, it is clear that bending strength increases with density and that this increase has a linear trend. Bending strength varied between under 5 MPa for the lowest density boards to over 340 MPa for the ones with the highest density. MOE behavior followed a similar trend to bending strength ranging from 200 MPa to 5000 MPa. Similar results were presented before by several authors with different materials. For example, De Melo et al. [19] studied the board density effect on the physical and mechanical properties of particleboards made from *Eucalyptus grandis* W. Hill ex Maiden, and they concluded that when density increased linearly from 0.6 g/cm^3^ to 0.8 g/cm^3^, both MOR and MOE increased. Kalaycioglu et al. [11] produced three-layer boards from Paulownia wood with 0.35 g/cm^3^ manufactured with densities of 0.55 g/cm^3^ and 0.65 g/cm^3^ and reported an increase in MOR, MOE and IB with the board density. Mechanical properties like bending strength and MOE seem to be directly proportional to density with high determination coefficients.

Internal bond increased with the density of the boards, similarly to bending strength and modulus of elasticity. Values ranged between 0.4 MPa and 0.7 MPa. Likewise, De Melo and Del Menezzi [19] obtained internal bond strength varying from around 0.25–0.4 MPa for densities of 0.6–0.8 g/cm^3^ for particleboards made from *Eucalyptus grandis*.

Contrary to the mechanical properties, water absorption decreased for higher-density boards. Water absorption ranged between 150% and 50%. Similar results were presented before by several author, such as, for instance, De Melo and Del Menezzi [19] for particleboards made from *Eucalyptus grandis* W. Hill ex Maiden and Cravo et al. [38] for particleboards based on cement packaging.

Thickness swelling has been reported to increase for higher-density boards. Khedari et al. [39], who studied a new insulating particleboard from durian peel and coconut coir, concluded that a higher density resulted in an increase of thickness swelling higher than the standard specification requirements for particleboards reaching about 29% and 35% for board density around 0.590 g/cm^3^ for Durian peel and coconut coir, respectively [39]. Similar results were presented by De Melo and Del Menezzi [19]. The same was observed in the present study, with 24 h thickness swelling increasing with density, as can be seen in Figure 12.

Moreover, the thermal conductivity of Paulownia particleboards increased with the board density, similar to what was observed before [56]. This has been attributed to the lower space and void for higher density particleboards, since the air in voids has a low thermal conductivity. Thermal conductivity ranged between 0.085–0.125 W/mK. The thermal conductivity was lower than was obtained before for a particleboard made with a mixture of durian peel and coconut coir (10:90) which was around 0.134 W/mK [56]. Nevertheless, this conductivity was obtained for boards with 0.856 g/cm^3^ density. If the comparison is made at a similar density, for instance around 0.600 g/cm^3^, durian peel and coconut coir have a lower thermal conductivity of 0.085 W/mK against 0.105 W/mK [56].

In accordance to NP EN 312 [46] the requirements for general purpose boards used in a dry environment (Type P1) are bending strength higher than 10.5 MPa for 8 mm thickness boards and 0.28 MPa for the internal bond. If these boards are to be used in furniture (Type P2), the requirements are 11 MPa for bending strength, 1800 MPa for MOE and 0.4 MPa for internal bond. Thickness swelling is only a requirement for boards used in a humid environment. For example, for non-structural boards, thickness swelling should be lower than 17% swelling in 24 h. Therefore, in order to fulfill the requirements for a dry environment, a density of around 0.65 g/cm^3^ is needed to achieve the necessary mechanical strength, and this would lead to a thermal conductivity of around 0.115 W/mK. If these boards were to be used in a humid environment, they would not fulfill the requirements because, to achieve a swelling under 17%, the maximum density would be around 50 g/cm^3^, and, for this density, mechanical properties would be lower than the minimum requirements. Bending strength would be around 5 MPa and MOE around 1000 Mpa. However, if no mechanical strength is needed, a lower thermal conductivity of around 0.085 W/mK could be obtained.

## 4. Conclusions

The main objective of this study was to produce particleboards from very young Paulownia trees with acceptable mechanical properties and lower thermal conductivity. The objectives were accomplished.

The specific conclusions of the study are:

1. Particleboards can be produced with density ranging from 0.39 g/cm^3^ to 0.92 g/cm^3^, bending strength 2–32 MPa and MOE 200–4900 MPa, Internal bond 0.4–1.6 MPa, water absorption 60–140%, thickness swelling 16–44% and thermal conductivity 0.085–0.125 W/mK.
2. In order to meet the requirements for a dry environment according to NP EN 312 standard, Paulownia particleboards need to have a density higher than 0.65 g/cm^3^ which leads to a thermal conductivity of around 0.115 W/mK.

## Data Availability

Data are available on request from the corresponding author.
